# Tumor necrosis factor-α synthesis inhibitor 3,6′-dithiothalidomide attenuates markers of inflammation, Alzheimer pathology and behavioral deficits in animal models of neuroinflammation and Alzheimer’s disease

**DOI:** 10.1186/1742-2094-9-106

**Published:** 2012-05-29

**Authors:** David Tweedie, Ryan A Ferguson, Kelly Fishman, Kathryn A Frankola, Henriette Van Praag, Harold W Holloway, Weiming Luo, Yazhou Li, Luca Caracciolo, Isabella Russo, Sergio Barlati, Balmiki Ray, Debomoy K Lahiri, Nigel H Greig, Susanna Rosi

**Affiliations:** 1 Laboratory of Neurosciences, Intramural Research Program, National Institute on Aging, National Institutes of Health, Baltimore, MD, 21224, USA; 2 Brain and Spinal Injury Center, University of California, San Francisco, CA, 94143, USA; 3 Molecular Neuroscience Unit, Brain Physiology and Metabolism Section, Intramural Research Program, National Institute on Aging, National Institutes of Health, Bethesda, MD, 20892, USA; 4 Division of Biology & Genetics, Department of Biomedical Sciences and Biotechnologies & National Institute of Neuroscience, University of Brescia, Brescia, 25123, Italy; 5 Laboratory of Molecular Neurogenetics, Department of Psychiatry, Institute of Psychiatric Research Indiana University School of Medicine, Indianapolis, IN, 46202, USA; 6 National Institute of Neurological Disorders and Stroke, National Institutes of Health, Bethesda, MD, 20892, USA; 7 Departments of Physical Therapy Rehabilitation Science and Neurological Surgery, University of California, San Francisco, CA, USA

**Keywords:** Neuroinflammation, Neurodegeneration, TNF-α, Neuroprotection, Alzheimer’s disease, Mild cognitive impairment, Amyoid-β peptide, Tau, Thalidomide, Lenalidomide

## Abstract

**Background:**

Neuroinflammation is associated with virtually all major neurodegenerative disorders, including Alzheimer’s disease (AD). Although it remains unclear whether neuroinflammation is the driving force behind these disorders, compelling evidence implicates its role in exacerbating disease progression, with a key player being the potent proinflammatory cytokine TNF-α. Elevated TNF-α levels are commonly detected in the clinic and animal models of AD.

**Methods:**

The potential benefits of a novel TNF-α-lowering agent, 3,6′-dithiothalidomide, were investigated in cellular and rodent models of neuroinflammation with a specific focus on AD. These included central and systemic inflammation induced by lipopolysaccharide (LPS) and Aβ_1–42_ challenge, and biochemical and behavioral assessment of 3xTg-AD mice following chronic 3,6′-dithiothaliodmide.

**Results:**

3,6′-Dithiothaliodmide lowered TNF-α, nitrite (an indicator of oxidative damage) and secreted amyloid precursor protein (sAPP) levels in LPS-activated macrophage-like cells (RAW 264.7 cells). This translated into reduced central and systemic TNF-α production in acute LPS-challenged rats, and to a reduction of neuroinflammatory markers and restoration of neuronal plasticity following chronic central challenge of LPS. In mice centrally challenged with Aβ_1–42_ peptide, prior systemic 3,6′-dithiothalidomide suppressed Aβ-induced memory dysfunction, microglial activation and neuronal degeneration. Chronic 3,6′-dithiothalidomide administration to an elderly symptomatic cohort of 3xTg-AD mice reduced multiple hallmark features of AD, including phosphorylated tau protein, APP, Aβ peptide and Aβ-plaque number along with deficits in memory function to levels present in younger adult cognitively unimpaired 3xTg-AD mice. Levels of the synaptic proteins, SNAP25 and synaptophysin, were found to be elevated in older symptomatic drug-treated 3xTg-AD mice compared to vehicle-treated ones, indicative of a preservation of synaptic function during drug treatment.

**Conclusions:**

Our data suggest a strong beneficial effect of 3,6′-dithiothalidomide in the setting of neuroinflammation and AD, supporting a role for neuroinflammation and TNF-α in disease progression and their targeting as a means of clinical management.

## Background

Neuroinflammation is a critical and invariable component of many neurodegenerative diseases. Indeed, the co-occurrence of inflammatory markers in plasma, cerebral spinal fluid (CSF) and post-mortem brain tissue has become a hallmark feature of Alzheimer’s disease (AD) and Parkinson’s disease (PD) [1-4]. This has given rise to the suggestion that inflammation within the CNS is an important contributing factor in the continuum of neurodegeneration, particularly in AD. The cytokine tumor necrosis factor-alpha (TNF-α) is a major mediator of inflammation that is commonly upregulated in biological samples from patients and animal models of human neurodegenerative disease [5-12]. In brain, TNF-α is primarily synthesized and released by glial cells that can be activated by trauma, infection or the presence of endogenous yet abnormal protein aggregates, such as amyloid-β (Aβ) peptides in AD. Furthermore, cytokines, including TNF-α, have been demonstrated to self-regulate immune/glial cells to increase their expression of amyloid-β precursor protein and Aβ in vitro [13]. Thus, in addition to the synthesis and release of cytotoxic factors, glial cells possess the ability, under appropriate conditions, to be an important non-neuronal, source of abnormal APP in brain, a hallmark feature in AD [14,15]. The existence of an altered brain microenvironment observed in neurodegenerative disease allows for and increases the likelihood of the generation of an unregulated immune response that potentiates any ongoing toxicity toward neurons.

In human post-mortem AD brain, a dramatic loss in neurons has been observed in the medial temporal lobe (hippocampus and entorhinal cortex). Over time, AD patients develop classical patterns of brain pathology, as described by Braak and Braak [16], and corresponding cognitive impairments [14]. Early stages of dementia may be associated with neuronal dysfunction causing the observed cognitive impairments rather than overt cell loss. It is possible that neuronal dysfunction is related to the presence of inflammatory factors, as rodent models of chronic neuroinflammation have been shown to induce impaired states of learning and memory in vivo [17,18]. In line with this, the expressions of genes associated with learning and memory have been reported to be altered by LPS administration in mice [19]. Additionally, disturbances in biochemical and functional correlates of learning at the cellular level, such as long-term potentiation (LTP) [20] and neural network activity [21], have been observed in models that mimic AD pathology. These findings support the concept that inflammation exacerbates the existing processes of neurodegeneration observed in AD.

It is plausible that the dysfunction of viable neurons during early stages of AD may not represent a permanent state and may thus be amenable to rescue, potentially providing improved outcomes for long-term nerve cell survival and function. Transgenic animal models are commonly used to investigate pathological processes of neurodegenerative diseases; one such model is the 3xTg-AD mouse model [22]. Data from 3xTg-AD mice suggest a clear involvement of cytokines, particularly TNF-α at pre-symptomatic stages of AD pathology [23]. Additionally, the use of this model has shown that in some circumstances neurons express TNF-α gene products [24], and that reduced TNF-α signaling [25] and microglia activation [26] mitigated disease progression.

Herein, to further define the role of TNF-α in neuroinflammation, neuronal dysfunction and cognitive impairment, we utilized a TNF-α synthesis-lowering agent, 3,6′-dithiothalidomide, developed within our laboratory [27]. This agent has been shown to effectively lower the levels of TNF-α and nitrite, a surrogate of nitric oxide metabolism, in LPS-treated macrophage-like cells in vitro [28], to reverse established hippocampus-dependent cognitive deficits induced by chronic neuroinflammation [29], as well as to reverse learning and memory behavioral deficits in a rodent model of head trauma [30]. We therefore assessed the biochemical and behavioral actions of 3,6′-dithiothalidomide in three models of neuroinflammation and in 3xTg-AD mice to evaluate TNF-α as a neurological drug target in AD.

## Methods and materials

### Pharmacological interventions

3,6′-Dithiothalidomide was prepared in 100% tissue culture grade dimethyl sulfoxide (DMSO, Sigma-Aldrich, St Louis, MO) for cell culture experiments, or as a suspension in a 1% carboxy methyl cellulose/saline solution (Fluka 21901). A concentration of 56 mg/kg body weight of drug was used for animal work unless stated otherwise. Lipopolysaccharide (LPS) was obtained from *Escherichia coli* (E coli) serotype 055:B5 (Sigma-Aldrich). Aβ_1–42_ or Aβ_42–1_ peptides were from American Peptide, Sunnyvale, CA.

### Cell culture

Mouse RAW 264.7 cells were purchased from ATCC (Manassas, VA, USA) and were grown in DMEM media supplemented with 10% FCS, penicillin 100 U/ml and streptomycin 100 μg/ml, and were maintained at 37°C and 5% CO_2._ Cells were propagated as described by ATCC guidelines. RAW 264.7 cells were cultured as has been previously described [28,31]. Cells were challenged with concentrations of LPS as indicated, and 24 h later, conditioned media was harvested and analyzed for the quantification of secreted TNF-α protein, nitrite and APP levels. Cellular health was assessed by use of the CellTiter 96 AQueous One Solution Cell Proliferation Assay (Promega, Madison, WI).

### Acute animal LPS drug study

An in vivo assessment of the effects of 3,6′-dithiothalidomide on the biosynthesis of LPS-induced TNF-α mRNA and protein was undertaken. The levels of hippocampal mRNA, plasma and CNS protein were determined. Male Fisher 344 rats (3 months of age) were challenged with LPS (1 mg/kg body weight, via the i.p. route). A series of blood samples were taken from the rats over a 5-h time period: [−60, 0 (LPS challenge), 30, 60, 90, 120, 180 and 240 min post LPS], plasma was generated from blood by conventional means. After 240 min the CNS was harvested, and all samples were immediately frozen to −70°C and stored for analyses.

### Chronic intracerebroventricularly animal LPS drug study

The rodents used for these experiments where male Fisher 344 rats (3 months of age). Four study groups were utilized: (1) artificial cerebrospinal fluid (aCSF) plus drug vehicle (aCSF-veh; *n* = 4); (2) aCSF plus 3,6′-dithiothalidomide (aCSF-DT; *n* = 6); (3) LPS plus drug vehicle (LPS-veh; *n* = 5) and (4) LPS plus 3,6′-dithiothalidomide (LPS-DT; *n* = 7). The LPS or aCSF were infused directly into the brain via an intracerebroventricular (i.c.v.) catheter (placement coordinates: 2.5 mm posterior to lambda, on the mid-line and 7.0 mm ventral to the dura) into the lateral ventricle as previously described [17,18,21,29]. Animals received daily i.p. administration of 3,6′-dithiothalidomide (56 mg/kg) or vehicle for 14 days starting the day of the surgery. On day 14 after surgery, each animal was placed in an open field and allowed to explore for 10 min. The open field environment consisted of a circular chamber (130 cm in diameter, 25 cm high) containing four different objects in the center. Total distance moved and time spent in different zones of the chamber were recorded with EthoVison XT software from Noldus (Noldus, Nijmegen Area, The Netherlands). All animals were euthanized by anesthesia in an isoflurane chamber followed by decapitation immediately after exploration. To ensure that transcription induced by euthanasia would not be detectable, the brain was quickly removed (between 120–150 s) and flash frozen in −80°C ice cold isopentane [17,18,21,29]. The fresh frozen brains were stored at −70°C until processing for in situ hybridization and fluorescence immunostaining as previously described [17,18,21,29].

### Acute intracerebroventricular Aβ_1–42_ peptide animal drug study

Adult male C57BL/6 mice (3 months of age) were utilized in this study. Mice received 3,6′-dithiothalidomide (56 mg/kg, i.p.) or vehicle daily for 14 days; after 7 days of treatment with drug animals were challenged with Aβ peptide. Aβ_1–42_ and the reverse peptide Aβ_42–1_ were reconstituted in phosphate-buffered saline (pH 7.4) and aggregated by incubation at 37°C for 7 days prior to administration. Aβ_1–42_ and Aβ_42–1_ (400 pmol) were then infused i.c.v. into the lateral ventricle as previously described [32].

### 3xTg-AD animal drug study

Adult and old male 3xTg-AD mice (10 and 17 months of age) were maintained on a 12-h light/dark cycle with free access to water and food. Animals received administration of either 3,6′-dithiothalidomide (42 mg/kg) or the vehicle i.p., daily for 6 weeks. Four to five weeks after the initiation of drug treatment, the animals were tested in the Morris Water Maze to assess acquisition (learning) and retention of spatial memory. After the completion of the Morris Water Maze assessment, the animals were euthanized by decapitation while under isoflurane anesthesia. The brain was immediately removed, and specific regions were excised and instantly frozen to allow later quantification of levels of various cortical proteins of interest: soluble Aβ_1–42_, total tissue APP, tau and phospho-tau protein, and the presynaptic proteins: SNAP25 and synaptophysin.

All animal studies were undertaken according to protocols approved by the respective Institutional Animal Care and Use Committee’s of the Intramural Research Program, National Institute on Aging (331-LNS-2012, 293-LNS-2013 and NICHD # 08–011), and the University of California (AN082537-03A), in compliance with the guidelines for animal experimentation of the National Institutes of Health (Department of Health, Education, and Welfare publication 85–23, revised, 1995).

### Quantitative RT-PCR for rat TNF-α mRNA

Total mRNA was isolated from the hippocampus using the RNeasy RNA isolation kit (Qiagen, Valencia, CA). Various concentrations of total mRNA extracted from rat brain were prepared for the generation of absolute and relative standard curves. The RNA samples and serial dilutions for standard curves were reverse-transcribed (Applied Biosystems), and PCR reactions were carried out using primers and probe sets purchased from Applied Biosystems (Foster City, CA): the TNF-α primers and probe set recognize exon 2–3 of TNF-α (assay location 391, GenBank accession NM_012675.2), and the GAPDH primers and probe set recognize exon 3 (assay location 295, GenBank accession NM_017008.3). The signals from the amplified PCR products were detected using the ABI Prism 7700 Sequence Detection System (Applied Biosystems), and obtained Ct values were calculated to the relative amount of RNA from the standard curves for each RNA transcript.

### ELISA analysis

TNF-α levels were measured by species-specific ELISAs; Mouse RAW 264.7 cell culture media TNF-α protein were measured with a mouse ELISA, BioLegend, Mouse TNF-α ELISA MAX™ Deluxe. Rat plasma and CNS protein was measured with a rat ELISA, BD OptEIA Rat TNF ELISA Kit II, BD Biosciences or an Ultrasensitive rat TNF-α, Invitrogen, respectively. Soluble human Aβ_1–42_, levels were measured by use of a human Aβ_42_ ELISA kit from Invitrogen. For all ELISA measurements, samples were assayed in duplicate, and the appropriate procedures were followed according to the manufacturer’s instructions.

### Immunohistochemistry

The brains of animals that received i.c.v. administered LPS or Aβ peptides were processed as previously described for the appropriate procedure [17,18,21,29,32]. For rat i.c.v. LPS studies, tissue was labeled with the following primary antibodies: MHC class II marker OX-6 (Pharmigen, San Diego, CA) for activated microglia and glial fibrillary acidic protein (GFAP; Pharmigen) for activated astrocytes; nuclei were counterstained with Sytox-Green (molecular Probes, Eugene, OR). Arc mRNA detection was performed as previously described [17,18,21]. Activated microglia numbers were quantified in the DG and CA3 area as previously described [18,29]. For mouse i.c.v. Aβ peptide studies activated microglia were identified and labeled with rabbit anti-CD11b (1:200; Chemicon, Temecula, CA). Fuoro-Jade B, a fluorochrome, was used to aid in the quantification of degenerating neurons in the dentate gyrus [33,34]. For mouse 3xTg-AD studies activated microglial cells in the subiculum and CA1 region were visualized using an anti-CD68 (Serotec) primary antibody.

### Morris water maze test

Spatial learning and memory were assessed using the Morris Water Maze. The target platform was submerged 1 cm below the water surface and placed at the midpoint of one quadrant. Visual cues were placed around the tank to orient the mice. The acquisition training sessions took place over 4 days (for i.c.v. Aβ peptide-injected mice, 3 days after Aβ administration) or over 6 days (for 3xTg-AD mice, 4 to 5 weeks after initiation of drug treatment). The memory retention assessments were performed at 24 h (Aβ peptide-injected mice) or at 4 and 24 h (3xTg-AD mice) after the last training session. The variables of interest were mouse reference memory, the time spent in the platform area and the number of platform crossings. These events were recorded and analyzed to determine age- and drug-related differences between the groups.

### Western blotting

Secreted APP levels were measured by Western blotting of equal volumes of harvested culture media after separation in a 10% Bis-Tris protein gel and probed with an antibody that recognizes all forms of APP (22 C11; Millipore, Billerica, MA). For 3xTg-AD mouse studies protein from each sample was separated by electrophoresis in Criterion gels (BioRad), then the transferred proteins were probed for APP (clone 6E10, Covance, Emeryville, CA), phospho and non-phospho tau (Thermo Scientific, Rockford, IL); SNAP 25 (Millipore), and synaptophysin (Santa Cruz Biotechnology, Santa Cruz, CA). Protein signals were obtained by chemiluminescence substrate methods, and signals were normalized to β-actin (GE/Amersham) [35,36].

### Statistical analysis

GraphPad Prism software was used to perform the statistical analysis of the following variables: plasma and CNS TNF-α levels; hippocampus TNF-α mRNA; Morris Water Maze platform escape latency; time spent in quadrants and platform crossings. A one-way ANOVA was performed on each group followed by a Bonferroni’s post test or a Fisher’s post-hoc test, where appropriate. For two-group comparisons, unpaired Student’s t-tests were carried out. One-way ANOVA was performed for variables measured from RAW 264.7 cells, TNF-α, nitrite and sAPP, and were followed by Bonferroni’s post hoc tests, where appropriate. For the immunostaining analysis, the control and experimental groups were the independent variable, and the percentages of neurons expressing Arc or activated microglia from various categories were the dependent variables. When an ANOVA was significant (*p* < 0.001), individual between-group comparisons were performed with Bonferroni’s post hoc tests to correct for multiple comparisons. Statistical analyses are provided in each figure legend and, where appropriate, involved one- or two-tailed t-tests for specific comparisons.

## Results

### 3,6′-Dithiothalidomide attenuates the synthesis of inflammatory mediators in vitro and in vivo

**Figure 1:**
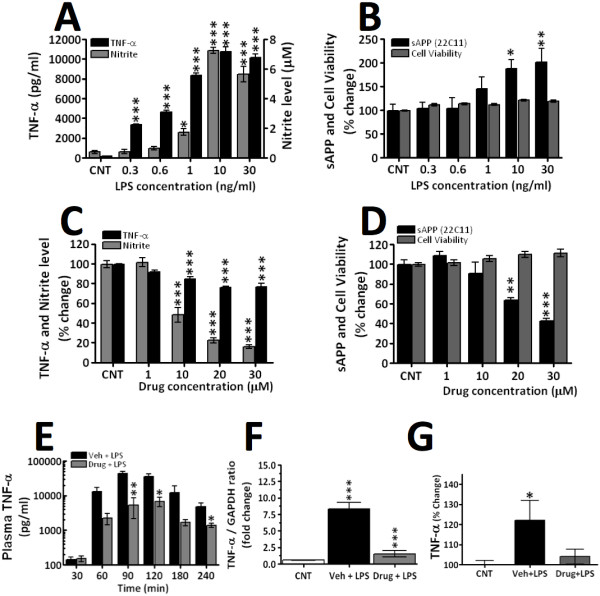
**Pretreatment with 3,6′-dithiothalidomide significantly attenuates LPS-induced elevations in TNF-α in vitroand in vivo. (A)** Increasing concentration of LPS in RAW 264.7 cells induced a dose-dependent elevation in TNF-α and nitrite levels. **(B)** In the same cells, LPS induced increases in media sAPP levels (*n* = 3); no cell toxicity was observed. **(C)** Pretreatment of cells with 3,6′-dithiothalidomide lowered the levels of TNF-α and nitrite-induced by LPS (*n* = 3–4). **(D)** Likewise, in the absence of toxicity the drug reduced the levels of sAPP. **(E)** The effects of intraperitoneal administration of LPS ± pretreatment with 3,6′-dithiothalidomide on rat plasma TNF-α levels over a 4-h time course are shown. LPS induced a marked elevation of plasma TNF-α protein (*n* = 7–9), which was significantly reduced by drug (*n* = 4–5). **(F)** Compared to control (*n* = 6), at 4 h, there was a marked LPS-induced increase in the levels of hippocampus TNF-α mRNA (*n* = 9). Pretreatment with drug significantly attenuated the LPS-induced expression of TNF-α mRNA (*n* = 5). **(G)** In line with LPS-induced systemic elevations in TNF-α, CNS TNF-α levels were likewise significantly elevated. 3,6′-Dithiothalidomide fully ameliorated this increase and restored CNS TNF-α levels to baseline. Data are expressed as mean ± SEM of *n* observations; levels of statistical significance are indicated as follows: *P < 0.05, **P < 0.01, ***P < 0.001.

The lipophilic, small molecular weight (mw: 290), TNF-α synthesis-lowering agent 3,6′-dithiothalidomide [27] was utilized in a series of studies aimed at assessing the role of TNF-α on neuroinflammation directed at an AD-like phenotype. RAW 264.7 cells treated with LPS recapitulate aspects of microglial cells observed in neurodegenerative diseases exemplified by AD. A challenge of RAW 264.7 cells with increasing concentrations of LPS induced a dose-dependent generation of TNF-α protein (Figure 1A), nitrite an intermediate of nitric oxide metabolism (Figure 1A) and the secretion of amyloid precursor protein (sAPP) into culture media, suggestive of an upregulated processing of APP (Figure 1B). These changes occurred without loss of cell viability (Figure 1B). Pretreatment of cells with 3,6′-dithiothalidomide prior to LPS (10 ng/ml) administration reduced the synthesis of each factor in a dose-dependent manner (Figure 1C and D), likewise without loss of cell viability (Figure 1D). The action of 3,6′-dithiothalidomide was additionally assessed in an acute in vivo rodent LPS model. Rats challenged with systemic LPS (i.p.) displayed a marked time-dependent increase in plasma TNF-α protein that was significantly attenuated by pretreatment with the agent (Figure 1E). Notably within the CNS, hippocampal TNF-α mRNA levels (Figure 1F) were elevated by LPS and markedly suppressed by drug treatment, as were TNF-α protein levels (Figure 1G).

### 3,6′-Dithiothalidomide treatment reduces LPS-induced chronic neuroinflammation and restores LPS-mediated abnormal hippocampal neuronal plasticity

**Figure 2:**
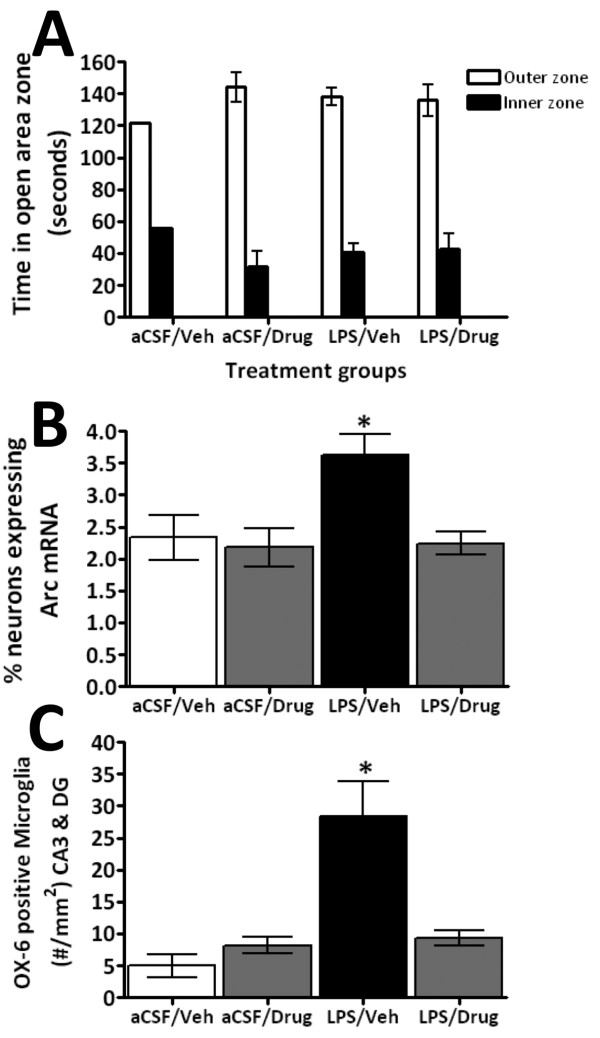
**Fourteen days after chronic intracerebroventricular infusion of LPS in rats, an altered state of neuronal plasticity and an elevated number of OX-6 positive microglial cells were observed in the hippocampus. Co-administration of 3,6′-dithiothalidomide suppressed these effects of LPS. (A)** After 14 days co-administration of LPS (i.c.v. [40]) with 3,6′-dithiothalidomide (i.p), no differences in the time spent in the outer and inner zone of an exploration chamber were observed between the treatment groups. In each animal cohort the rodents displayed a preference for the outer zone rather than the inner zone. The mean time spent in the two zones over the first 3 min of a 10-min exploratory period is shown (aCSF + Veh *n* = 1; *n* = 4–9). **(B)** The effects of the 10-min behavioral assessment on the immediate early gene. Activity regulated cytoskeletal protein (*Arc*) gene expression in the upper blade of the hippocampal neurons is presented; *in situ* hybridization data indicate that there was an increase in the number of neurons expressing *Arc* mRNA in LPS + vehicle-treated animals. The behavior-LPS-induced elevation was prevented by treatment with 3,6′-dithiothalidomide (*n* = 4–9). See Figure 3 for representative images of Arc staining. **(C)** LPS administration induced a significant increase in numbers of OX-6 positive microglial cells, while treatment with 3,6′-dithiothalidomide prevented this effect of LPS. See Figure 4 for representative images of activated microglia staining. Data are expressed as mean ± SEM of *n* observations; levels of statistical significance are indicated as follows: *P < 0.05.

**Figure 3:**
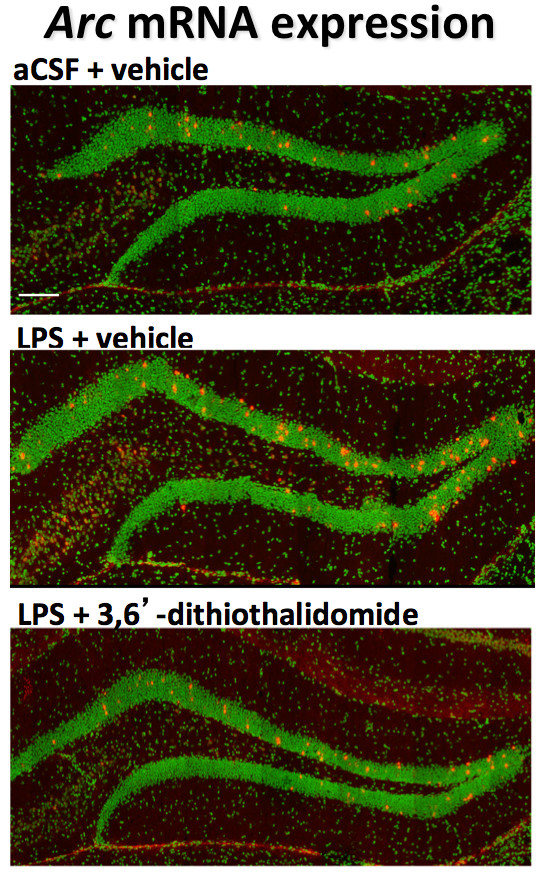
**3,6′-Dithiothalidomide (56 mg/kg i.p.) treatment attenuates the aberrant expression of Arc mRNA induced by intercerebroventricular administration of LPS after a uniform level of behavioral activity**. Representative flat images of neurons labeled for Arc mRNA (*red*) within the DG, after behavioral exploration of a novel environment for 10 min. Nuclei are counterstained in *green*. The *upper panel* illustrates the numbers of Arc positive (*+ve*) cells in animals infused with aCSF and treated with drug vehicle. The *middle panel* displays the numbers of Arc + ve cells in LPS-infused, vehicle-treated animals. The *lower panel* shows the numbers of Arc + ve cells in LPS-infused, drug-treated animals. The *scale bar* indicates 100 μm.

**Figure 4:**
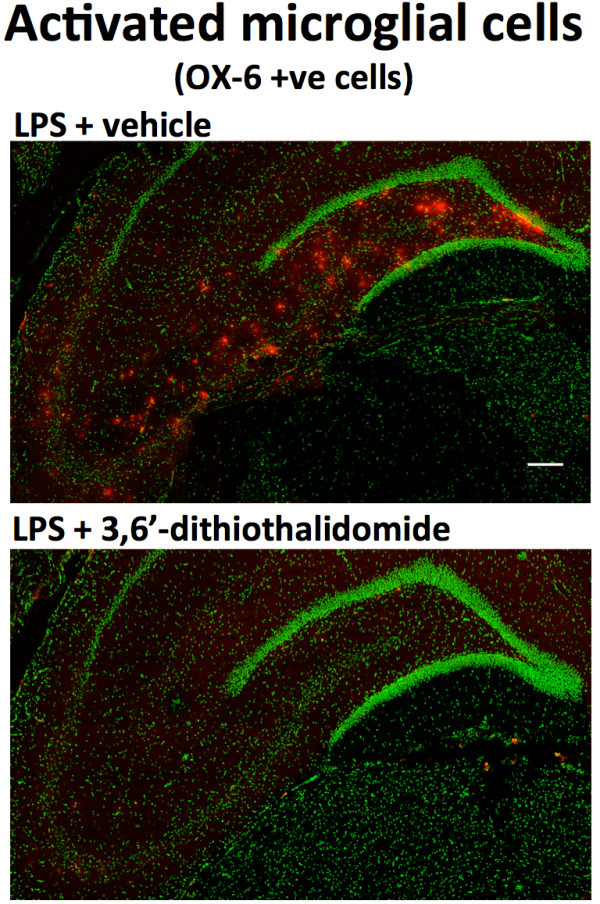
**3,6′-Dithiothalidomide (56 mg/kg i.p.) treatment suppresses the activation of microglial cells induced by the intercerebroventricular administration of LPS.** Representative flat images of staining for activated microglial cells in the dentate gyrus (*DG*) and CA3 areas of rat hippocampus are shown. The *upper panel* illustrates high numbers of activated microglial cells after treatment with LPS. The *lower panel* illustrates that drug treatment attenuates the numbers of LPS-induced activated microglia. Microglia are stained *red*; nuclei are counterstained *green*. The scale bar indicates 100 μm.

**Figure 5:**
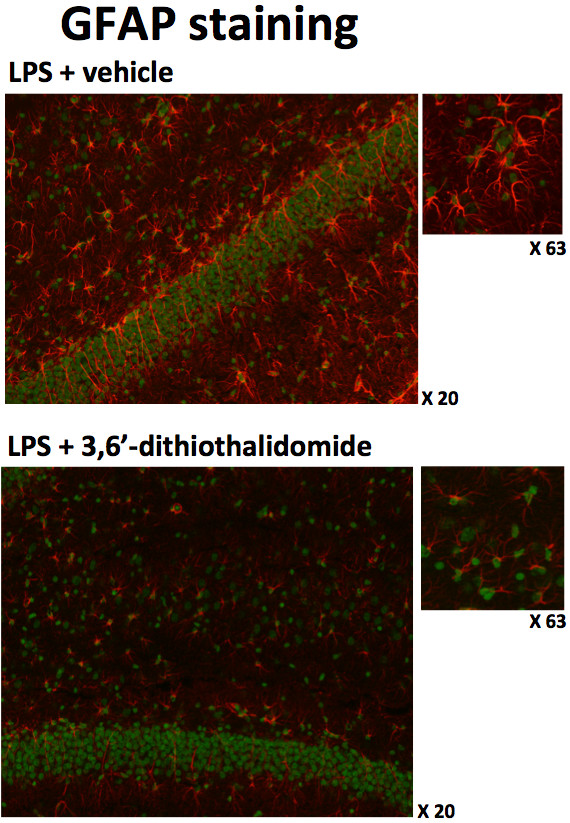
**3,6′-Dithiothalidomide (56 mg/kg i.p.) treatment reduces the activation of astrocyte cells induced by the intercerebroventricular administration of LPS.** Representative images of GFAP + ve cells in a field of view of the granule cell layer of the hippocampus are shown. The *upper left panel* (×20 objective magnification for both treatments) illustrates the highly activated numbers and morphology of astrocytes after the administration of LPS; the smaller image is a higher magnification of a section from the same image (×63 objective magnification, for both treatments). The *lower panel* illustrates how drug treatment markedly reduces the activated morphology of astrocytes after treatment with LPS; similarly to the above, this effect is further illustrated in the higher magnification side image. Astrocytes are stained *red*, whereas nuclei are counterstained *green.*

The potential benefits of lowering TNF-α levels on neuroinflammation-induced altered neuroplasticity were assessed. To recreate the hostile brain microenvironment associated with chronic neuroinflammation, we utilized the properties associated with direct chronic CNS infusion of a small quantity of LPS in rats. This infusion, with and without systemic 3,6′-dithiothalidomide treatment, was well tolerated. When animals were allowed to explore an open field arena for 10 min, no differences were observed in either speed or exploration pattern (Figure 2A), indicating that such treatments had no impact on levels of anxiety or motility of animals. However, chronic i.c.v. LPS did induce a significant increase in the number of granule cells expressing the plasticity-related immediate early gene (IEG) Arc within the suprapyramidal blade of the dentate gyrus (Figures 2B and 3) compared to aCSF animals. No differences were observed within the infrapyramidal blade (not shown). The IEG Arc is required for synaptic plasticity and is essential for memory processing [37,38]; behaviorally induced Arc expression within the hippocampus is significantly modified by chronic inflammation [39]. Notably, the centrally mediated LPS-induced disturbances in Arc expression were abolished by systemically administered 3,6′-dithiothalidomide. In parallel with Arc mRNA levels, the number of OX-6 positive (MHC class II) microglial cells was markedly elevated in LPS-treated animals, whereas drug treatment prevented this rise (Figures 2C and 4). Furthermore, GFAP immunofluorescence staining indicated a marked upregulation in both the number and activated morphology of astrocytes in response to LPS treatment; these effects likewise were significantly attenuated by 3,6′-dithiothalidomide (Figure 5).

### 3,6′-Dithiothalidomide treatment attenuates the effects of central administration of toxic Aβ_1–42_ peptide on behavior, cell viability and microglia activation

**Figure 6:**
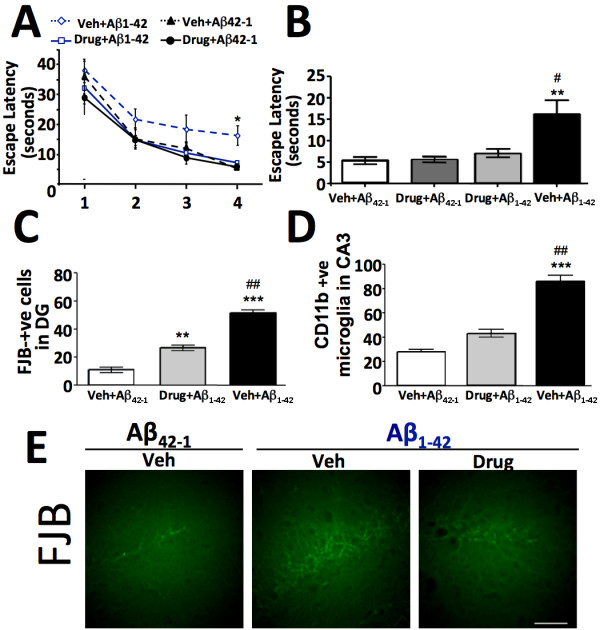
**3,6′-Dithiothalidomide (56 mg/kg i.p.) prevented Aβ**_**1–42**_**peptide-induced memory defects, neuronal cell degeneration and associated elevated CD11b staining of microglial cells.** After 7 days of drug administration, aggregated Aβ_1–42_ peptide was administered directly into the CNS (and reverse peptide Aβ_42–1_ was utilized as a control) (for aggregation/aging process, see [32,41]). Mouse memory function was assessed by the Morris Water Maze; escape latencies measured over a 4-day trial period **(A)** and the final assessment undertaken 24 h after the last training session are shown (**B***n* = 8). **(C)** After the completion of behavioral assessments mice were euthanized, and the levels of neuronal degeneration were indicated by determining the level of Fluoro-Jade B labeling in the dentate gyrus; Aβ_1–42_ peptide induced a significant degree of dye incorporation that was attenuated by 3,6′-dithiothalidomide (*n* = 3–4). **(D)** Numbers of CD11b-positive cells in the CA3 hippocampus region were determined to be elevated only in the vehicle + Aβ_1–42_ group (*n* = 3–4). **(E)** Representative photomicrographs of Fluoro-Jade B-positive neurons in the dentate gyrus of control (vehicle-treated Aβ_42–1_-injected) mice, vehicle-treated Aβ_1–42_-injected mice and 3,6′-dithiothalidomide-treated Aβ_1–42_-injected mice. *Indicates comparisons with control (vehicle-treated Aβ_42–1_-injected) mice; #indicates comparisons with 3,6′-dithiothalidomide-treated Aβ_1–42_-injected mice (*n* = 3–4). Data are expressed as mean ± SEM of *n* observations; levels of statistical significance are indicated as follows: * or #*P* < 0.05, ** or ##*P* < 0.01, *** or ###*P* < 0.001.

The direct administration of aged Aβ peptide, allowing its oligomerization, into the CNS of wild-type (wt) mice, was undertaken to emulate the inflammatory microenvironment of the AD brain. In our study, a single i.c.v. administration of Aβ_1–42_ was undertaken 7 days after the initiation of a daily schedule of systemic (i.p.) 3,6′-dithiothaliomide, utilizing a dose determined to be effective to ameliorate LPS-induced CNS elevations in TNF-α in the prior studies. Control animals were administered reverse peptide (with or without similar 3,6′-dithiothalidomide treatment). As illustrated in Figures 6A and B, Aβ_1–42_ alone induced a marked deficit in the learning ability of mice in the Morris Water Maze paradigm (for both acquisition and retention). This was accompanied by neuronal degeneration (assessed by Fluoro-Jade B staining) and an increased presence of CD11b-positive staining microglial cells within the dentate gyrus (DG) of the same animals (Figure 6C, D and E). Treatment of mice with 3,6′-dithiothalidomide, prior to Aβ_1–42_, markedly mitigated the actions of this toxic peptide. Specifically, drug-treated animals performed at a level similar to control mice in the Morris Water Maze, and showed evidence of reduced levels of both neuronal degeneration and CD11b positive microglial cells in comparison to Aβ_1–42_ alone mice. No differences were evident between mice administered Aβ_42–1_ with or without drug treatment in any of the measured parameters.

### 3,6′-Dithiothaliomide, administered daily for 6 weeks, normalized age-associated biochemical, cellular and behavioral features of AD observed in the 3xTg-AD mouse model

Mice containing three transgenes (APP_Swe_ + PS1_M146V_ + tau_P301L_) associated with AD [15] were utilized to assess the effects of 3,6′-dithiothalidomide on two distinct age groups of animals, of 10 and 17 months age at study onset, referred to here as adult and old, respectively. These two age ranges were selected based on prior studies [36,42] demonstrating that the line of 3xTg-AD mice utilized in our study, which had been backcrossed onto a C57BL/6 background for seven generations, presented with brain AD pathology at 16 months age. Our chosen age groups hence can be considered to be prior to and post the onset of evident significant AD pathology, and these 3xTg-AD mice were administered 3,6′-dithiothalidomide or vehicle for 6 weeks thereafter.

**Figure 7:**
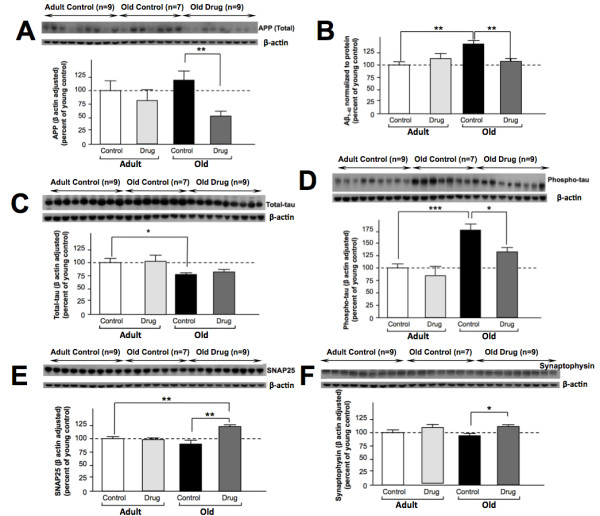
**3,6′-Dithiothalidomide (42 mg/kg i.p. once daily × 6 weeks) attenuated age-associated changes in hallmark features of AD in old 3xTg-AD animals.** Two cohorts of 3xTg-AD mice, adult and old, were evaluated after a 6-week regimen with daily administration of 3,6′-dithiothalidomide or vehicle. After completion of the Morris Water Maze assessment animals were euthanized, and the levels of hallmark features of Alzheimer’s disease were assessed. **(A)** Treatment with drug (*n* = 9) significantly reduced the levels of total APP in the old animals vs. old control animals (*n* = 7). **(B)** Cortical soluble Aβ_1–42_ levels were increased in the old + control animals compared to younger adult + control (*n* = 7–9, *P* < 0.01). Drug treatment reduced the soluble Aβ_1–42_ in the old mice (*n* = 8), but not in younger adult ones. **(C)** Levels of total tau protein were lower in old control animals compared to younger adult control mice; however, drug treatment had no effect on total tau levels in either age group. **(D)** Phosphorylated tau protein levels were elevated in old animals compared to younger adult animals (*n* = 8–9). Treatment of the old animals with drug reduced levels of phosphorylated tau protein. The levels of SNAP 25 **(E)** and synaptophysin **(F)** were elevated in old drug-treated mice (*P* < 0.01 and *P* < 0.05, both *n* = 7–9, respectively), compared to old vehicle animals. Data are presented in percent change from appropriate control terms and are expressed as mean ± SEM of *n* observations; levels of statistical significance are indicated as follows: *P < 0.05, **P < 0.01, ***P < 0.001.

Following the final assessment of mouse learning and memory, animals were euthanized and various brain biochemical measures were undertaken. As assessed in the cerebral cortex, a trend to an elevated level in total APP (6E10) was evident when comparing adult vehicle with old vehicle animals (Figure 7A). 3,6′-Dithiothalidomide proved to be well tolerated by 3xTg-AD mice, and lowered total APP levels by 19% and 43% in adult and old mice compared to their respective vehicle controls (Figure 7A). Examination of soluble Aβ_1–42_ levels indicated a marked age effect (Figure 7B), with old vehicle controls expressing levels 143% of adult ones. This elevation was fully inhibited by 3,6′-dithiothalidomide, whose levels were similar to adult control and drug-treated mice (Figure 7B). Total tau protein levels were reduced (23%) in old vehicle animals compared to adult ones, and were unaffected by drug treatment (Figure 7C). However, levels of phosphorylated tau protein presented a strong age-associated rise (176% of adult controls) that, similarly to soluble Aβ_1–42_ protein, was attenuated by drug treatment in the older group (Figure 7D). Interestingly, the levels of two synaptic markers, SNAP25 and synaptophysin, which showed a trend to decline in the old vehicle control group (90% and 93% of respective adult vehicle levels) were found to be significantly elevated (19% and 37%, respectively) in old drug-treated mice compared to old vehicle ones (Figures 7E and F).

**Figure 8:**
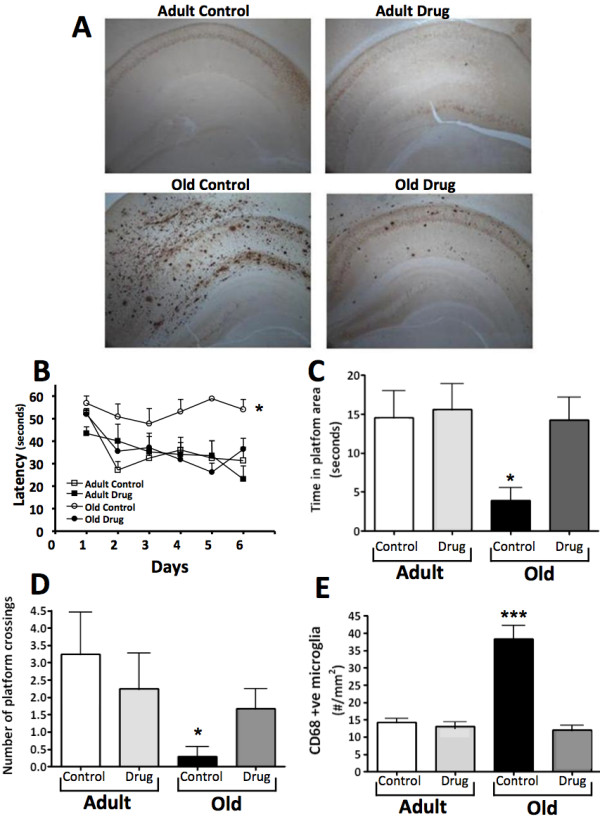
**3,6′-Dithiothalidomide (42 mg/kg i.p. once daily × 6 weeks) attenuated age-associated Aβ plaque deposition, memory deficits and indices of neuroinflammation in the subiculum and CA1 region of the hippocampus in the elder of two cohorts of 3xTg-AD mice. (A)** Male 3xTg-AD mice displayed an age-dependent increase in Aβ plaque deposition; drug treatment reduced the numbers of Aβ plaques in the older (old) animals. *Upper panel*: in younger adult (*adult*) animals (approximately 11.5 months of age at the time of death) few if any Aβ plaques were detectable by immunohistochemical methods. However, in old animals (approximately 18.5 months of age at the time of death) male 3xTg-AD mice presented marked deposition of Aβ plaques that were markedly reduced by drug treatment. Representative brain sections illustrating the levels of Aβ plaque formation detected in the adult and old animals and the effects of 6 weeks of 3,6′-dithiothalidomide on plaques are shown. **(B)** Morris Water Maze training over 6 days with four trials per day revealed a significant interaction between age and treatment (F(1,33) = 4.77; *P* < 0.0360). In addition a main effect of treatment was observed F(1,33) = 5.34; *P* < 0.0272 (*n* = 7–9); specific comparisons showed that drug treatment attenuated the learning deficits observed in the old group. **(C and D)** Probe trial performed 4 h after the last training session on day 6 showed an interaction between age and drug treatment F(1,33) = 4.60; *P* < 0.0393. Treatment: F(1,33) = 4.26; *P* < 0.0468. This indicated that drug-treated old mice were able to remember the location of the submerged platform more akin to that of the adult mice, age and treatment for time in target zone: F(1,33) = 4.60; *P* < 0.0393; treatment: F(1,33) = 4.26; *P* < 0.0468. **(E)** The numbers of CD68 positive microglial cells were significantly elevated in the old vehicle-treated mice compared to the drug-treated old and both sets of adult mice. Data are expressed as mean ± SEM of *n* observations; levels of statistical significance are indicated as follows: *P < 0.05, **P < 0.01, ***P < 0.001.

Immunohistochemical staining of insoluble Aβ plaques indicated marked plaque formation only in the old 3xTg-AD mice; this Aβ plaque staining was dramatically reduced by drug treatment (Figure 8A). Memory function, assessed by the Morris Water Maze, illustrated a deficit in learning and acquisition of the location of the hidden platform in old vehicle control mice during training (Figure 8B and C). Probe trial data obtained 4 h following their final training session indicated that these old vehicle control animals failed to remember the location of the platform, as illustrated by the low time spent within the target zone as well as by the low number of platform crossings (Figure 8C and D). 3,6′-Dithiothalidomide abolished this age-associated memory deficit (Figure 8B, C and D), with treated mice performing on par with adult vehicle control and drug-treated animals.

The levels of CD68-positive microglial cells within the subiculum and CA1 brain region were quantified as a marker of the inflammatory microenvironment in the hippocampus, as these regions are among those showing the highest concentration of Aβ plaque staining (Figure 8A). CD68-positive microglial cells were significantly elevated in number (three-fold) only within old vehicle control mice, and this rise was fully abolished by the drug (Figure 8E). Hence, treatment with 3,6′-dithiothalidomide induced a marked normalization of key biochemical, learning and memory features of AD in old 3xTg-AD mice.

## Discussion

Here we demonstrate that the TNF-α-lowering agent, 3,6′-dithiothalidomide, ameliorates key aspects of neuroinflammation in multiple acute and longer term CNS rodent models. Importantly, several of these models emulate specific cardinal characteristics of AD, and highlight the complex cyclic interaction among the synthesis of TNF-α, the development of neuroinflammation and impact on disease progression, inducing its advancement. Our data suggest that breaking this cycle by lowering TNF-α generation and neuroinflammation can favorably impact AD, as assessed at both a behavioral and biochemical level, even late during the disease course. Our studies hence reinforce the significant role of neuroinflammation in AD and other degenerative neurological disorders, and highlight the potential for targeting TNF-α.

TNF-α has been implicated in the pathogenesis of a wide number of neurological disorders, including AD, PD, stroke and head trauma [5-12]. Indeed, TNF-α levels have been found to be elevated within the CSF of AD patients by as much as 25-fold [43], in line with substantial elevations in TNF-α synthesis that were rapidly induced in RAW 264.7 cells and animals challenged with LPS (Figure 1A,E,F,G). Studies in subjects with mild cognitive impairment (MCI) that progress to develop AD suggest that increased CSF TNF-α levels are an early event, and their rise correlates with disease progression [44]. In accord with this, Janelsins and colleagues [23] noted an elevated expression of TNF-α transcripts within the entorhinal cortex of 3xTg-AD mice at 2 months, prior to the appearance of amyloid and tau pathology, and this increase correlated with the onset of cognitive deficits in these mice [45]. These studies, together with others demonstrating that (1) TNF-α polymorphisms that elevate TNF-α production may increase AD risk, particularly in patients carrying one or more apolipoprotein E ϵ4 alleles [46-49], and that (2) genetic ablation of TNF-α receptor 1 (TNFR1) in APP23 AD mice [50] or a selective lowering of soluble TNF-α brain levels in 3xTg-AD mice [25] reduces AD progression, support the concept of TNF-α inhibition strategies for treatment of AD [10,51,52].

Protein-based TNF-α inhibitors (etanercept and infliximab) that can effectively regulate circulating TNF-α levels by binding them [53] have provided a means to initially target brain TNF-α in AD, and perispinal etanercept administration followed by Trendelenburg positioning in a small prospective open-label pilot study has been reported to provide a rapid onset of cognitive improvement [54]. TNF-α levels can also be regulated at the level of synthesis, which is tightly controlled at the level of mRNA stability to facilitate rapid responses to exogenous and endogenous stimuli [55], as occurs with LPS and Aβ challenge, respectively, and hence is amenable to regulation by small-molecular-weight drugs. The presence of adenylate-uridylate-rich elements (AREs) within the 3′-UTR of TNF-α mRNA supports the potential for post-transcriptional repression, targeting it for rapid degradation or inhibition of translation. This is mediated through interactions with RNA-binding proteins (RBPs), epitomized by HuR, which binds and stabilizes ARE-containing transcripts and conveys them to translational machinery to upregulate protein synthesis and, conversely, by tristetraprolin, which aids the acceleration and degradation of bound mRNAs [56-59].

Exogenous signals, as arise from exposure to bacterial proteins, potently induce inflammatory responses within the CNS in a manner mimicked by RAW 264.7 cells challenged with LPS [28]. The resulting elevation in TNF-α derives from an LPS-mediated increase in the half-life of TNF-α mRNA, allowing release of its translational repression. By contrast, translational blockade can be induced by small-molecular-weight compounds, such as thalidomide, which induce a shortening of the TNF-α mRNA half-life [55]. In this regard, 3,6′-dithiothalidomide is a more potent TNF-α-lowering thalidomide analogue that acts at the level of the 3′UTR of TNF-α [27,60]. As is evident in Figure 1, LPS challenge to RAW 264.7 cells or animals activated toll-like receptors (TLR), and induced the generation of TNF-α and nitrite, a stable surrogate marker of highly unstable nitric oxide production. APP levels also were elevated, in line with prior studies [61] that additionally describe rises in interleukin (IL)-1, 6 and 12, and cyclooxygenase-2 [62]. 3,6′-Dithiothalidomide dose-dependently lowered LPS-induced TNF-α, nitrite and APP levels in the absence of cellular toxicity in RAW 264.7 cells, in contrast to thalidomide, which proved ineffective at concentrations up to 30 μM (not shown), but has been reported to lower APP levels in PC12 and SH-SY5Y neuronal cell lines [63]. This action of 3,6′dithiothalidomide effectively translated to both a systemic LPS challenge in vivo, lowering systemic and central TNF-α expression (Figure 1E-G), as well as to a central LPS challenge.

Administered to brain, LPS reliably induces chronic neuroinflammation associated with the activation of microglia, which is allied to impaired hippocampal-dependent spatial cognitive function [17,18,21]. In the present study this was achieved by the slow continuous infusion of a low dose of LPS into the fourth ventricle of the brain, which produced microglia activation within the hippocampus and, importantly, induced abnormal Arc expression in response to a simple behavioral task (Figures 2 and 3). The activity-regulated, cytoskeleton-associated IEG Arc is a key regulator of protein synthesis-dependent forms of synaptic plasticity, which are fundamental to memory formation [37,64,65]. In healthy brain, Arc protein functions in a transient manner, and its abnormally elevated sustained expression, as occurs during neuroinflammation, may generate synaptic noise and thereby impair long-term memory formation [64]. Co-administration of systemic 3,6′-dithiothalidomide, which has been reported to readily enter the brain (brain/plasma ratio 1.34) [30] and reversed an acute LPS-mediated increase in TNF-α expression (Figure 1G), fully inhibited LPS-induced activation of microglia and the resulting altered coupling of neural activity with de novo synthesis of Arc (Figures 4 and 3, respectively). A similar dose of 3,6′-dithiothalidomide has recently been described to normalize the expression of Arc and to restore the acquisition and consolidation of spatial memory impairments in a fully established model of neuroinflammation [29], in which LPS was administered to rodents for a full 28 days prior to drug treatment (rather than in parallel with drug treatment, as in the present study). In this study by Belarbi et al. [29], 3,6′-dithiothalidomide normalized LPS-induced elevations in brain TNF-α expression, but not IL-1β, and additionally normalized the expression of specific genes involved within the TLR-mediated signaling pathways (in particular, TLR2, TLR4, Hmgb1 and IRAK1) that are established to lead to elevated TNF-α expression [66].

Aβ, particularly in the form of soluble oligomeric assemblies or Aβ-derived diffusible ligands (ADDLs) [67-69], has been described to target synapses, induce neuronal dysfunction and impair cognition. Its administration into the lateral ventricle of mice has been widely used to model neuroinflammation and induce these AD-related impairments [32,70-72], which in the present study resulted in the activation of microglia and neuronal degeneration within the DG, accompanied by a learning impairment in the Morris Water Maze paradigm (Figure 6). Pretreatment of animals with 3,6′-dithiothalidomide markedly inhibited each of these aspects and, together with our prior studies, suggested that the agent could prove of value in Tg models of AD that, like the human condition, increasingly develop neuroinflammation during disease progression [23,24]. This hypothesis was tested in two cohorts of 3xTg-AD mice of 10 and 17 months age, chosen to represent times that in our specific line coincided with the pre- and post-development of amyloid plaques and neurofibrillary tangles, as the presence of activated microglia in close proximity to amyloid plaques is a cardinal feature of AD-afflicted brain [73].

The pre-pathological upregulation of TNF-α and associated enhancement of activated microglia have been reported in the 3xTg-AD mouse model [23,25], and it has been postulated that these activated immune cells are key in the process of clearing extracellular Aβ [62]. A potential consequence of heightened Aβ exposure, however, is microglia TLR4 stimulation and a resultant upregulation of cytokine production and release [74]. TNF-α as well as IL-1β can correspondingly elevate Aβ generation by stimulating γ-secretase activity [24,75], potentially spawning a self-propagating positive feedback loop of Aβ induction of inflammation and TNF-α signaling that, in turn, may provoke further Aβ generation [5,6,9,10,62]. In our study, in accord with the literature [24], activated microglia were markedly elevated in old versus adult vehicle-treated 3xTg-AD mice (Figure 8E), which additionally presented with a significant elevation in brain Aβ_1–42_ and phosphorylated tau levels, a decline in total tau and a trend towards elevation of APP levels (Figure 7). A substantial accumulation of extracellular amyloid plaques was clearly evident within the cerebral cortex and hippocampus of old versus adult 3xTg-AD mice, which was accompanied by deficits in learning and memory, as assessed within the Morris Water Maze paradigm (Figure 8). The administration of 3,6′-dithiothalidomide to old 3xTg-AD mice reversed each of these parameters, significantly reducing Aβ_1–42_, phosphorylated tau and APP levels, lowering levels of activated microglia and fully ameliorating memory deficits (Figures 7 and 8), which were accompanied by an elevation in synaptic protein markers (Figures 7E and F). These drug-induced changes are in line with studies by McAlpine and colleagues [25], demonstrating that blockade of TNF-α signaling (either by viral vector-mediated expression of TNFR constructs or by crossing 3xTg-AD mice with TNFR1 knockout mice) significantly suppressed AD pathology. Importantly, our studies additionally demonstrate that cognitive deficits that accompany the classical pathology of AD appear to be reversible, at least in the 3xTg-AD mouse model.

A caveat with this 3xTg-AD mouse model, like all such models, is that it provides a partial model of the human disease. APP and tau expressions (specifically, human APP_Swe_ and human tau_P301L_) are driven in the 3xTg-AD model by the unnatural mouse Thy1.2 regulatory element [22]. Hence, the possibility that some actions of 3,6′-dithiothalidomide may be mediated via suppression of this unnatural transgene promoter cannot be ruled out. Importantly, however, the action of 3,6′-dithiothalidomide to favorably lower APP levels as well as neuroinflammation in cellular studies (Figure 1) occurred in cells controlled by their natural endogenous regulatory elements, and wt rodents were used in all other studies.

TNF-α has been shown to regulate numerous cellular processes, not only inflammation and cell death, but also cellular differentiation and survival, and achieves this by binding and activating two cognate receptors, TNFR1 (p55) and TNFR2 (p75) [76]. TNFR1, expressed ubiquitously including on neurons, astrocytes and microglia, possesses an intracellular death domain and contributes to neuronal dysfunction and death following activation by soluble TNF-α [77], whereas TNFR2, principally expressed on cells of hematopoietic origin but also on neurons, has been associated with cell survival [76,78-80] and chiefly responds to membrane-bound TNF-α [81,82]. The engagement of homotrimeric TNF-α to either receptor can activate three major signaling pathways: an apoptotic cascade initiated via the TNF-α receptor-associated death domain, a nuclear factor kappa B (NFκB) signaling pro-survival pathway implemented via NFκB-mediated gene transcriptional actions, and a JNK (c-Jun N-terminal kinase) cascade involved in cellular differentiation and proliferation that is generally pro-apoptotic [62]. In large part, although the contrasting pro-survival versus death-inducing actions of TNF-α plausibly rely on the TNF-α receptor subtype activation, the target cell types involved and their expression ratio of TNFR1/2 and associated coupling proteins, the temporal levels of available soluble and membrane-bound TNF-α [79], and the scale and duration of neuroinflammation combine in determining the eventual physiological consequences of TNF-α receptor activation [5,6,9,10,62]. Consequent to the diverse actions of TNF-α and the influence of the brain microenvironment in which they occur, it is hence not always clear under which conditions TNF-α promotes beneficial versus deleterious neuronal actions, and this, in large part, accounts for how an initially pro-survival response may develop into a pro-apoptotic one.

Under appropriate conditions TNF-α signaling, primarily via TNFR2, can mediate homeostatic actions, epitomized by its role in AMPA receptor surface expression and synaptic scaling to impact LTP [83], as well as neuroprotective ones [9,10]. The genetic ablation of TNFR1 and -R2 in 3xTg-AD mice has been described to increase the progression of AD pathology [84]. Furthermore, TNF-α has a reported role in hippocampal development and function [85] and, with the expression of both TNFR1 and -R2 on neuronal progenitor cells, it can modulate neurogenesis within the hippocampal neurons under pathological conditions [86-89]. The finding that adult 3xTg-AD mice were not detrimentally impacted by 3,6′-dithiothalidomide suggests that such homeostatic actions of TNF-α signaling were largely unimpaired, although clearly substantial classical preclinical toxicological studies are warranted before the agent can be considered for clinical use.

## Conclusion

In synopsis, the present data demonstrate the key role of TNF-α in acute and chronic neuroinflammatory models that have relevance to neurodegeneration and, in particular, to AD and its progression. The TNF-α synthesis inhibitor 3,6′-dithiothalidomide, administered at doses that compare favorably to those of thalidomide in human studies (100 to 1,200 mg daily [90]), appears to advantageously reset the delicate balance between the pro- versus anti-apoptotic actions of this signaling cascade in the brain. This resulted in an inhibition of neuroinflammation and reduced AD progression in 3xTg-AD mice, and thereby supports the feasibility of targeting TNF-α as a potential treatment strategy for AD and other neurological disorders involving a neuroinflammatory component.

## Abbreviations

AREs, adenylate-uridylate-rich elements; AD, Alzheimer’s disease; ADDLs, amyloid-β-derived diffusible ligands; Aβ, amyloid-β peptide; aCSF, artificial cerebrospinal fluid; CSF, cerebral spinal fluid; CNS, central nervous system; DG, dentate gyrus; DMSO, dimethyl sulfoxide; GFAP, glial fibrillary acidic protein; IEG, immediate early gene; IL, interleukin; IP, interperitoneal; ICV, intracerebroventricularly; JNK, c-Jun N-terminal kinase; LPS, lipopolysaccharide; LTP, long-term potentiation; MCI, mild cognitive impairment; MW, molecular weight; NFκB, nuclear factor kappa B; PD, Parkinson’s disease; RBPs, RNA-binding proteins; sAPP, secreted amyloid precursor protein; TNF-α, tumor necrosis factor-alpha; TNFR1, TNF-α receptor 1; TNFR2, TNF-α receptor 2; TLR, Toll-like receptors; WT, wild type.

## Misc

Debomoy K Lahiri Francesca Bosetti, Nigel H Greig and Susanna Rosi equal contributors

## Competing interests

NHG and HWH declare that they are co-inventors of the original 3,6′-dithiothalidomide patent. Having assigned all their rights to the US government, they declare that they have no ownership, financial interest or any other competing interests. All other authors declare no competing interests.

## Authors’ contributions

DT contributed to the design of the study, and undertook experimental studies and biochemical analyses in Figures 1, 7 and 8, and the associated statistical analyses. RAF and KF contributed to the experimental studies and analyses in Figures 2, 3, 4 and 5. KAF, HVP, HWH and YL contributed to the experimental design, and experimental studies and analyses in Figures 1, 7 and 8. WL undertook synthetic chemistry, chemical characterization and stability assessments. LC, IR, SB and FB contributed to the experimental design and undertook experimental studies and analyses in Figure 6. BR and DKL undertook tissue preparations, Western blot and ELISA analyses in Figure 7. FB, DKL, SR and NHG contributed to the study conception and design, and experimental studies, and wrote the manuscript. All the authors read and approved the final manuscript.

## References

[B1] McGeer PL Itagaki S Boyes BE McGeer EG Reactive microglia are positive for HLA-DR in the substantia nigra of Parkinson’s and Alzheimer’s disease brains Neurology 1988 38 1285 1291 10.1212/WNL.38.8.1285 3399080

[B2] Hirsch EC Hunot S Damier P Faucheux B Glial cells and inflammation in Parkinson’s disease: a role in neurodegeneration? Ann Neurol 1988 44 S115 S120 9749582 10.1002/ana.410440717

[B3] Nagatsu T Mogi M Ichinose H Togari A Cytokines in Parkinson’s disease J Neural Transm Suppl 2000 58 143 151 11128604

[B4] Liu B Hong JS Role of microglia in inflammation-mediated neurodegenerative diseases: mechanisms and strategies for therapeutic intervention J Pharmacol Exp Ther 2003 304 1 7 10.1124/jpet.102.035048 12490568

[B5] Tweedie D Sambamurti K Greig NH TNF-α inhibition as a treatment strategy for neurodegenerative disorders: New drug candidates and targets Curr Alzheimer Res 2007 4 378 385 10.2174/156720507781788873 17908040

[B6] Frankola KA Greig NH Luo W Tweedie D Targeting TNF-α to elucidate and ameliorate neuroinflammation in neurodegenerative diseases CNS Neurol Disord Drug Targets 2011 10 391 403 10.2174/187152711794653751 21288189 PMC4663975

[B7] Hirsch EC Breidert T Rousselet E Hunot S Hartmann A Michel PP The role of glial reaction and inflammation in Parkinson’s disease Ann NY Acad Sci 2003 991 214 228 12846989 10.1111/j.1749-6632.2003.tb07478.x

[B8] Sawada M Imamura K Nagatsu T Role of cytokines in inflammatory process in Parkinson’s disease J Neural Transm Suppl 2006 70 373 381 10.1007/978-3-211-45295-0_57 17017556

[B9] Tansey MG Frank-Cannon TC McCoy MK Lee JK Martinez TN McAlpine FE Ruhn KA Tran TA Neuroinflammation in Parkinson’s disease: is there sufficient evidence for mechanism-based interventional therapy? Front Biosci 2008 13 709 717 10.2741/2713 17981581

[B10] McCoy MK Tansey MG TNF signaling inhibition in the CNS: implications for normal brain function and neurodegenerative disease J Neuroinflammation 2008 5 45 10.1186/1742-2094-5-45 18925972 PMC2577641

[B11] Watters O O’Connor JJ A role for tumor necrosis factor-α in ischemia and ischemic preconditioning J Neuroinflammation 2011 8 87 10.1186/1742-2094-8-87 21810263 PMC3161872

[B12] Hallenbeck JM The many faces of tumor necrosis factor in stroke Nat Med 2002 8 1363 1368 10.1038/nm1202-1363 12457181

[B13] Lahiri DK Chen D Vivien D Ge YW Greig NH Rogers JT Role of cytokines in the gene expression of amyloid beta-protein precursor: identification of a 5′-UTR-binding nuclear factor and its implications in Alzheimer’s disease J Alzheimers Dis 2003 5 81 90 12719626 10.3233/jad-2003-5203

[B14] Sambamurti K Greig NH Lahiri DK Advances in the cellular and molecular biology of the beta-amyloid protein in Alzheimer’s disease Neuromolecular Med 2002 1 1 31 10.1385/NMM:1:1:1 12025813

[B15] Sastre M Richardson JC Gentleman SM Brooks DJ Inflammatory risk factors and pathologies associated with Alzheimer’s disease Curr Alzheimer Res 2011 8 132 141 21345169 10.2174/156720511795256062

[B16] Braak H Braak E Neuropathological staging of Alzheimer-related changes Acta Neuropathol 1991 82 239 259 10.1007/BF00308809 1759558

[B17] Rosi S Ramirez-Amaya V Vazdarjanova A Worley PF Barnes CA Wenk GL Neuroinflammation alters the hippocampal pattern of behaviorally induced Arc expression J Neurosci 2005 25 723 731 10.1523/JNEUROSCI.4469-04.2005 15659610 PMC6725337

[B18] Rosi S Vazdarjanova A Ramirez-Amaya V Worley PF Barnes CA Wenk GL Memantine protects against LPS-induced neuroinflammation, restores behaviorally-induced gene expression and spatial learning in the rat Neuroscience 2006 142 1303 1315 10.1016/j.neuroscience.2006.08.017 16989956

[B19] Bonow RH Aïd S Zhang Y Becker KG Bosetti F The brain expression of genes involved in inflammatory response, the ribosome, and learning and memory is altered by centrally injected lipopolysaccharide in mice Pharmacogenomics J 2009 9 116 126 10.1038/tpj.2008.15 18957951 PMC2728029

[B20] Hauss-Wegrzyniak B Lynch MA Vraniak PD Wenk GL Chronic brain inflammation results in cell loss in the entorhinal cortex and impaired LTP in perforant path-granule cell synapses Exp Neurol 2002 176 336 341 10.1006/exnr.2002.7966 12359175

[B21] Rosi S Ramirez-Amaya V Vazdarjanova A Esparza EE Larkin PB Fike JR Wenk GL Barnes CA Accuracy of hippocampal network activity is disrupted by neuroinflammation: rescue by memantine Brain 2009 132 2464 2477 10.1093/brain/awp148 19531533 PMC2732266

[B22] Oddo S Caccamo A Shepherd JD Murphy MP Golde TE Kayed R Metherate R Mattson MP Akbari Y LaFerla FM Triple-transgenic model of Alzheimer’s disease with plaques and tangles: intracellular Abeta and synaptic dysfunction Neuron 2003 39 409 421 10.1016/S0896-6273(03)00434-3 12895417

[B23] Janelsins MC Mastrangelo MA Oddo S LaFerla FM Federoff HJ Bowers WJ Early correlation of microglial activation with enhanced tumor necrosis factor-alpha and monocyte chemoattractant protein-1 expression specifically within the entorhinal cortex of triple transgenic Alzheimer’s disease mice J Neuroinflammation 2005 2 23 10.1186/1742-2094-2-23 16232318 PMC1276812

[B24] Janelsins MC Mastrangelo MA Park KM Sudol KL Narrow WC Oddo S LaFerla FM Callahan LM Federoff HJ Bowers WJ Chronic neuron-specific tumor necrosis factor-alpha expression enhances the local inflammatory environment ultimately leading to neuronal death in 3xTg-AD mice Am J Pathol 2008 173 1768 1782 10.2353/ajpath.2008.080528 18974297 PMC2626388

[B25] McAlpine FE Lee JK Harms AS Ruhn KA Blurton-Jones M Hong J Das P Golde TE LaFerla FM Oddo S Blesch A Tansey MG Inhibition of soluble TNF signaling in a mouse model of Alzheimer’s disease prevents pre-plaque amyloid-associated neuropathology Neurobiol Dis 2009 34 163 177 10.1016/j.nbd.2009.01.006 19320056 PMC2948857

[B26] Fonseca MI Ager RR Chu SH Yazan O Sanderson SD LaFerla FM Taylor SM Woodruff TM Tenner AJ Treatment with a C5aR antagonist decreases pathology and enhances behavioral performance in murine models of Alzheimer’s disease J Immunol 2009 183 1375 1383 10.4049/jimmunol.0901005 19561098 PMC4067320

[B27] Zhu X Giordano T Yu Q-S Holloway HW Perry T Lahiri DK Brossi A Greig NH Thiothalidomides: Novel isosteric analogs of thalidomide with enhanced TNF-α inhibitory activity J Med Chem 2003 46 5222 5229 10.1021/jm030152f 14613324

[B28] Tweedie D Frankola KA Luo W Li Y Greig NH Thalidomide analogues suppress lipopolysaccharide-induced synthesis of TNF-α and nitrite, an intermediate of nitric oxide, in a cellular model of inflammation Open Biochem J 2011 5 37 44 10.2174/1874091X01105010037 21792375 PMC3141331

[B29] Belarbi K Jopson T Tweedie D Arellano C Luo W Greig NH Rosi S TNF-alpha protein synthesis inhibitor restores neuronal function and reverses cognitive deficits induced by chronic neuroinflammation J Neuroinflammation 2012 9 23 10.1186/1742-2094-9-23 22277195 PMC3298520

[B30] Baratz R Tweedie D Rubovitch V Luo W Yoon JS Hoffer BJ Greig NH Pick CG Tumor necrosis factor-α synthesis inhibitor, 3,6′-dithiothalidomide, reverses behavioral impairments induced by minimal traumatic brain injury in mice J Neurochem 2011 118 1032 1042 10.1111/j.1471-4159.2011.07377.x 21740439 PMC3397686

[B31] Tweedie D Luo W Short RG Brossi A Holloway HW Li Y Yu QS Greig NH A cellular model of inflammation for identifying TNF-alpha synthesis inhibitors J Neurosci Methods 2009 183 182 187 10.1016/j.jneumeth.2009.06.034 19583982 PMC2756970

[B32] Choi SH Bosetti F Cyclooxygenase-1 null mice show reduced neuroinflammation in response to beta-amyloid Aging 2009 1 234 244 20157512 10.18632/aging.100021 PMC2806008

[B33] Schmued LC Hopkins KJ Fluoro-Jade: novel fluorochromes for detecting toxicant-induced neuronal degeneration Toxicol Pathol 2000 28 91 99 10.1177/019262330002800111 10668994

[B34] El Khoury J Toft M Hickman SE Means TK Terada K Geula C Luster AD Ccr2 deficiency impairs microglial accumulation and accelerates progression of Alzheimer-like disease Nat Med 2007 13 432 438 10.1038/nm1555 17351623

[B35] Bailey JA Ray B Greig NH Lahiri DK Rivastigmine lowers Aβ and increases sAPPα levels, which parallel elevated synaptic markers and metabolic activity in degenerating primary rat neurons PLoS One 2011 6 7 e21954 10.1371/journal.pone.0021954 21799757 PMC3142110

[B36] Li Y Duffy KB Ottinger MA Ray B Bailey JA Holloway HW Tweedie D Perry T Mattson MP Kapogiannis D Sambamurti K Lahiri DK Greig NH GLP-1 receptor stimulation reduces amyloid-beta peptide accumulation and cytotoxicity in cellular and animal models of Alzheimer’s disease J Alzheimers Dis 2010 19 1205 1219 20308787 10.3233/JAD-2010-1314 PMC2948479

[B37] Guzowski JF Lyford GL Stevenson GD Houston FP McGaugh JL Worley PF Barnes CA Inhibition of activity-dependent arc protein expression in the rat hippocampus impairs the maintenance of long-term potentiation and the consolidation of long-term memory J Neurosci 2000 20 3993 4001 10818134 10.1523/JNEUROSCI.20-11-03993.2000 PMC6772617

[B38] Plath N Ohana O Dammermann B Errington ML Schmitz D Gross C Mao X Engelsberg A Mahlke C Welzl H Kobalz U Stawrakakis A Fernandez E Waltereit R Bick-Sander A Therstappen E Cooke SF Blanquet V Wurst W Salmen B Bösl MR Lipp HP Grant SG Bliss TV Wolfer DP Kuhl D Arc/Arg3.1 is essential for the consolidation of synaptic plasticity and memories Neuron 2006 52 437 444 10.1016/j.neuron.2006.08.024 17088210

[B39] Rosi S Neuroinflammation and the plasticity-related immediate-early gene Arc Brain Behav Immun 2011 1 Suppl S39 S49 21320587 10.1016/j.bbi.2011.02.003 PMC3098296

[B40] Rosi S Ramirez-Amaya V Hauss-Wegrzyniak B Wenk GL Chronic brain inflammation leads to a decline in hippocampal NMDA-R1 receptors J Neuroinflammation 2004 1 12 10.1186/1742-2094-1-12 15285803 PMC500869

[B41] Maurice T Lockhart BP Privat A Amnesia induced in mice by centrally administered beta-amyloid peptides involves cholinergic dysfunction Brain Res 1996 706 181 193 10.1016/0006-8993(95)01032-7 8822355

[B42] Hirata-Fukae C Li HF Hoe HS Gray AJ Minami SS Hamada K Niikura T Hua F Tsukagoshi-Nagai H Horikoshi-Sakuraba Y Mughal M Rebeck GW LaFerla FM Mattson MP Iwata N Saido TC Klein WL Duff KE Aisen PS Matsuoka Y Females exhibit more extensive amyloid, but not tau, pathology in an Alzheimer transgenic model Brain Res 2008 1216 92 103 18486110 10.1016/j.brainres.2008.03.079

[B43] Tarkowski E Blennow K Wallin A Tarkowski A Intracerebral production of tumor necrosis factor-alpha, a local neuroprotective agent, in Alzheimer disease and vascular dementia J Clin Immunol 1999 19 223 230 10.1023/A:1020568013953 10471976

[B44] Tarkowski E Andreasen N Tarkowski A Blennow K Intrathecal inflammation precedes development of Alzheimer’s disease J Neurol Neurosurg Psychiatry 2003 74 1200 1205 10.1136/jnnp.74.9.1200 12933918 PMC1738668

[B45] Billings LM Oddo S Green KN McGaugh JL LaFerla FM Intraneuronal Abeta causes the onset of early Alzheimer’s disease-related cognitive deficits in transgenic mice Neuron 2005 45 675 688 10.1016/j.neuron.2005.01.040 15748844

[B46] Perry RT Collins JS Wiener H Acton R Go RC The role of TNF and its receptors in Alzheimer’s disease Neurobiol Aging 2001 22 873 883 10.1016/S0197-4580(01)00291-3 11754994

[B47] Laws SM Perneczky R Wagenpfeil S Müller U Förstl H Martins RN Kurz A Riemenschneider M TNF polymorphisms in Alzheimer disease and functional implications on CSF beta-amyloid levels Hum Mutat 2005 26 29 35 10.1002/humu.20180 15895461

[B48] Ramos EM Lin MT Larson EB Maezawa I Tseng LH Edwards KL Schellenberg GD Hansen JA Kukull WA Jin LW Tumor necrosis factor alpha and interleukin 10 promoter region polymorphisms and risk of late-onset Alzheimer disease Arch Neurol 2006 63 1165 1169 10.1001/archneur.63.8.1165 16908746

[B49] Aleong R Blain JF Poirier J Pro-inflammatory cytokines modulate glial apolipoprotein E secretion Curr Alzheimer Res 2008 5 33 37 10.2174/156720508783884666 18288929

[B50] He P Zhong Z Lindholm K Berning L Lee W Lemere C Staufenbiel M Li R Shen Y Deletion of tumor necrosis factor death receptor inhibits amyloid-β generation and prevents learning and memory deficits in Alzheimer’s mice J Cell Biol 2007 178 829 841 10.1083/jcb.200705042 17724122 PMC2064547

[B51] Tobinick E Tumour necrosis factor modulation for treatment of Alzheimer's disease: rationale and current evidence CNS Drugs 2009 23 713 725 10.2165/11310810-000000000-00000 19689163

[B52] Tobinick E Deciphering the physiology underlying the rapid clinical effects of perispinal etanercept in Alzheimer’s disease Curr Alzheimer Res 2012 9 99 109 10.2174/156720512799015073 22191562

[B53] Lin J Ziring D Desai S Kim S Wong M Korin Y Braun J Reed E Gjertson D Singh RR TNF-α blockade in human diseases: an overview of efficacy and safety Clin Immunol 2008 126 13 30 10.1016/j.clim.2007.08.012 17916445 PMC2291511

[B54] Tobinick EL Gross H Rapid cognitive improvement in Alzheimer’s disease following perispinal etanercept administration J Neuroinflammation 2008 5 2 10.1186/1742-2094-5-2 18184433 PMC2211476

[B55] Moreira AL Sampaio EP Zmuidzinas A Frindt P Smith KA Kaplan G Thalidomide exerts its inhibitory action on tumor necrosis factor alpha by enhancing mRNA degradation J Exp Med 1993 177 1675 1680 10.1084/jem.177.6.1675 8496685 PMC2191046

[B56] Abdelmohsen K Kuwano Y Kim HH Gorospe M Posttranscriptional gene regulation by RNA-binding proteins during oxidative stress: implications for cellular senescence Biol Chem 2008 389 243 255 18177264 10.1515/BC.2008.022 PMC8481862

[B57] Stamou P Kontoyiannis DL Posttranscriptional regulation of TNF mRNA: a paradigm of signal-dependent mRNA utilization and its relevance to pathology Curr Dir Autoimmun 2010 11 61 79 20173387 10.1159/000289197

[B58] Patil CS Liu M Zhao W Coatney DD Li F VanTubergen EA D’Silva NJ Kirkwood KL Targeting mRNA stability arrests inflammatory bone loss Mol Ther 2008 16 1657 1664 10.1038/mt.2008.163 18682699 PMC2752748

[B59] Khera TK Dick AD Nicholson LB Mechanisms of TNFalpha regulation in uveitis: focus on RNA-binding proteins Prog Retin Eye Res 2010 29 610 621 10.1016/j.preteyeres.2010.08.003 20813201

[B60] Greig NH Giordano T Zhu X Yu QS Perry TA Holloway HW Brossi A Rogers JT Sambamurti K Lahiri DK Thalidomide-based TNF-alpha inhibitors for neurodegenerative diseases Acta Neurobiol Exp 2004 64 1 9 10.55782/ane-2004-1486 15190675

[B61] Mönning U Sandbrink R Banati RB Masters CL Beyreuther K Transforming growth factor beta mediates increase of mature transmembrane amyloid precursor protein in microglial cells FEBS Lett 1994 342 267 272 10.1016/0014-5793(94)80514-8 8150082

[B62] Park KM Bowers WJ Tumor necrosis factor-alpha mediated signaling in neuronal homeostasis and dysfunction Cell Signal 2010 22 977 983 10.1016/j.cellsig.2010.01.010 20096353 PMC2860549

[B63] Avramovich Y Amit T Youdim MB Non-steroidal anti-inflammatory drugs stimulate secretion of non-amyloidogenic precursor protein J Biol Chem 2002 277 31466 31473 10.1074/jbc.M201308200 12070143

[B64] Rosi S Neuroinflammation and the plasticity-related immediate-early gene Arc Brain Behav Immun 2011 25 Suppl 1 S39 S49 21320587 10.1016/j.bbi.2011.02.003 PMC3098296

[B65] Korb E Finkbeiner S Arc in synaptic plasticity: from gene to behavior Trends Neurosci 2011 34 591 598 10.1016/j.tins.2011.08.007 21963089 PMC3207967

[B66] Kawai T Adachi O Ogawa T Takeda K Akira S Unresponsiveness of MyD88- deficient mice to endotoxin Immunity 1999 11 115 122 10.1016/S1074-7613(00)80086-2 10435584

[B67] Shankar GM Li S Mehta TH Garcia-Munoz A Shepardson NE Smith I Brett FM Farrell MA Rowan MJ Lemere CA Regan CM Walsh DM Sabatini BL Selkoe DJ Amyloid-beta protein dimers isolated directly from Alzheimer’s brains impair synaptic plasticity and memory Nat Med 2008 14 837 842 10.1038/nm1782 18568035 PMC2772133

[B68] Ashe KH Zahs KR Probing the biology of Alzheimer’s disease in mice Neuron 2010 66 631 645 10.1016/j.neuron.2010.04.031 20547123 PMC2956420

[B69] Lacor PN Buniel MC Furlow PW Clemente AS Velasco PT Wood M Viola KL Klein WL Abeta oligomer-induced aberrations in synapse composition, shape, and density provide a molecular basis for loss of connectivity in Alzheimer’s disease J Neurosci 2007 27 796 807 10.1523/JNEUROSCI.3501-06.2007 17251419 PMC6672917

[B70] Yankner BA The pathogenesis of Alzheimer’s disease. Is amyloid beta-protein the beginning or the end? Ann N Y Acad Sci 2000 924 26 28 11193797 10.1111/j.1749-6632.2000.tb05555.x

[B71] Qiao X Cummins DJ Paul SM Neuroinflammation-induced acceleration of amyloid deposition in the APPV717F transgenic mouse Eur J Neurosci 2001 14 474 482 10.1046/j.0953-816x.2001.01666.x 11553297

[B72] Yamada M Chiba T Sasabe J Nawa M Tajima H Niikura T Terashita K Aiso S Kita Y Matsuoka M Nishimoto I Implanted cannula-mediated repetitive administration of Abeta25-35 into the mouse cerebral ventricle effectively impairs spatial working memory Behav Brain Res 2005 164 139 146 10.1016/j.bbr.2005.03.026 16122819

[B73] McGeer PL Itagaki S Tago H McGeer EG Reactive microglia in patients with senile dementia of the Alzheimer type are positive for the histocompatibility glycoprotein HLA-DR Neurosci Lett 1987 79 195 200 10.1016/0304-3940(87)90696-3 3670729

[B74] Jin JJ Kim HD Maxwell JA Li L Fukuchi K Toll-like receptor 4-dependent upregulation of cytokines in a transgenic mouse model of Alzheimer’s disease J Neuroinflammation 2008 5 23 10.1186/1742-2094-5-23 18510752 PMC2430555

[B75] Liao YF Wang BJ Cheng HT Kuo LH Wolfe MS Tumor necrosis factor-alpha, interleukin-1beta, and interferon-gamma stimulate gamma-secretase-mediated cleavage of amyloid precursor protein through a JNK-dependent MAPK pathway J Biol Chem 2004 279 49523 49532 10.1074/jbc.M402034200 15347683

[B76] Wajant H Pfizenmaier K Scheurich P Tumor necrosis factor signaling Cell Death Differ 2003 10 45 65 10.1038/sj.cdd.4401189 12655295

[B77] Grell M Wajant H Zimmermann G Scheurich P The type 1 receptor (CD120a) is the high-affinity receptor for soluble tumor necrosis factor Proc Natl Acad Sci USA 1998 95 570 575 10.1073/pnas.95.2.570 9435233 PMC18461

[B78] Fontaine V Mohand-Said S Hanoteau N Fuchs C Pfizenmaier K Eisel U Neurodegenerative and neuroprotective effects of tumor Necrosis factor (TNF) in retinal ischemia: opposite roles of TNF receptor 1 and TNF receptor 2 J Neurosci 2002 22 RC216 11917000 10.1523/JNEUROSCI.22-07-j0001.2002 PMC6758303

[B79] Yang L Lindholm K Konishi Y Li R Shen Y Target depletion of distinct tumor necrosis factor receptor subtypes reveals hippocampal neuron death and survival through different signal transduction pathways J Neurosci 2002 22 3025 3032 11943805 10.1523/JNEUROSCI.22-08-03025.2002 PMC6757531

[B80] Marchetti L Klein M Schlett K Pfizenmaier K Eisel UL Tumor necrosis factor (TNF)-mediated neuroprotection against glutamate-induced excitotoxicity is enhanced by N-methyl-D-aspartate receptor activation. Essential role of a TNF receptor 2-mediated phosphatidylinositol 3-kinase-dependent NF-kappa B pathway J Biol Chem 2004 279 32869 32881 10.1074/jbc.M311766200 15155767

[B81] Grell M Douni E Wajant H Löhden M Clauss M Maxeiner B Georgopoulos S Lesslauer W Kollias G Pfizenmaier K Scheurich P The transmembrane form of tumor necrosis factor is the prime activating ligand of the 80 kDa tumor necrosis factor receptor Cell 1995 83 793 802 10.1016/0092-8674(95)90192-2 8521496

[B82] Grell M Tumor necrosis factor (TNF) receptors in cellular signaling of soluble and membrane-expressed TNF J Inflammation 1995–1996 47 8 17 8913925

[B83] Stellwagen D Malenka RC Synaptic scaling mediated by glial TNF-alpha Nature 2006 440 7087 1054 1059 10.1038/nature04671 16547515

[B84] Montgomery SL Mastrangelo MA Habib D Narrow WC Knowlden SA Wright TW Bowers WJ Ablation of TNF-RI/RII expression in Alzheimer’s disease mice leads to an unexpected enhancement of pathology: implications for chronic pan-TNF-α suppressive therapeutic strategies in the brain Am J Pathol 2011 179 2053 2070 10.1016/j.ajpath.2011.07.001 21835156 PMC3181376

[B85] Golan H Levav T Mendelsohn A Huleihel M Involvement of tumor necrosis factor alpha in hippocampal development and function Cereb Cortex 2004 14 97 105 10.1093/cercor/bhg108 14654461

[B86] Bernardino L Agasse F Silva B Ferreira R Grade S Malva JO Tumor necrosis factor-alpha modulates survival, proliferation, and neuronal differentiation in neonatal subventricular zone cell cultures Stem Cells 2008 26 2361 2371 10.1634/stemcells.2007-0914 18583543

[B87] Iosif RE Ekdahl CT Ahlenius H Pronk CJ Bonde S Kokaia Z Jacobsen SE Lindvall O Tumor necrosis factor receptor 1 is a negative regulator of progenitor proliferation in adult hippocampal neurogenesis J Neurosci 2006 26 9703 9712 10.1523/JNEUROSCI.2723-06.2006 16988041 PMC6674454

[B88] Fioravanzo L Venturini M Liddo RD Marchi F Grandi C Parnigotto PP Folin M Involvement of rat hippocampal astrocytes in β-amyloid-induced angiogenesis and neuroinflammation Curr Alzheimer Res 2010 7 591 601 10.2174/156720510793499020 20704555

[B89] Belarbi K Arellano C Ferguson R Jopson T Rosi S Chronic neuroinflammation impacts the recruitment of adult-born neurons into behaviorally relevant hippocampal networks Brain Behav Immun 2012 26 18 23 10.1016/j.bbi.2011.07.225 21787860 PMC3221820

[B90] Eleutherakis-Papaiakovou V Bamias A Dimopoulos MA Thalidomide in cancer medicine Ann Oncol 2004 15 1151 1160 10.1093/annonc/mdh300 15277253

